# ANXA1sp Protects against Sepsis-Induced Myocardial Injury by Inhibiting Ferroptosis-Induced Cardiomyocyte Death via SIRT3-Mediated p53 Deacetylation

**DOI:** 10.1155/2023/6638929

**Published:** 2023-04-04

**Authors:** Song Qin, Yingcong Ren, Banghai Feng, Xiaoqin Wang, Junya Liu, Jie Zheng, Kang Li, Miao Chen, Tao Chen, Hong Mei, Xiaoyun Fu

**Affiliations:** ^1^Soochow University Medical College, Suzhou 215000, China; ^2^Department of Critical Care Medicine, Affiliated Hospital of Zunyi Medical University, Zunyi, Guizhou 563000, China; ^3^Department of Critical Care Medicine, Zunyi Hospital of Traditional Chinese Medicine, Zunyi, Guizhou 563000, China; ^4^Department of Pediatric, The Second Affiliated Hospital of Zunyi Medical University, Zunyi, Guizhou 563000, China

## Abstract

Sepsis-induced myocardial injury (SIMI), a common complication of sepsis, may cause significant mortality. Ferroptosis, a cell death associated with oxidative stress and inflammation, has been identified to be involved in SIMI. This study sought to investigate the role of ANXA1 small peptide (ANXA1sp) in SIMI pathogenesis. In this study, the mouse cardiomyocytes (H9C2 cells) were stimulated with lipopolysaccharide (LPS) to imitate SIMI *in vitro*. It was shown that ANXA1sp treatment substantially abated LPS-triggered H9C2 cell death and excessive secretion of proinflammatory cytokines (TNF-*α*, IL-1*β*, and IL-6). ANXA1sp pretreatment also reversed the increase of ROS and MDA generation as well as the decrease of SOD and GSH activity in H9C2 cells caused by LPS treatment. In addition, ANXA1sp considerably eliminated LPS-caused H9C2 cell ferroptosis, as revealed by the suppression of iron accumulation and the increase in GPX4 and FTH1 expression. Furthermore, the ameliorative effects of ANXA1sp on LPS-induced H9C2 cell damage could be partially abolished by erastin, a ferroptosis agonist. ANXA1sp enhanced SIRT3 expression in LPS-challenged H9C2 cells, thereby promoting p53 deacetylation. SIRT3 knockdown diminished ANXA1sp-mediated alleviation of cell death, inflammation, oxidative stress, and ferroptosis of LPS-treated H9C2 cells. Our study demonstrated that ANXA1sp is protected against LPS-induced cardiomyocyte damage by inhibiting ferroptosis-induced cell death via SIRT3-dependent p53 deacetylation, suggesting that ANXA1sp may be a potent therapeutic agent for SIMI.

## 1. Introduction

Sepsis, a severe systemic inflammatory response syndrome featured with multiorgan dysfunction [1], mainly arises from excessive host response to infection [2]. Approximately, half of the sepsis patients develop myocardial injury [3], known as sepsis-induced myocardial injury (SIMI), which is associated with increased mortality [4]. In addition, SIMI, in turn, contributes to multiorgan dysfunction in sepsis [5]. As a result of a poor understanding of SIMI pathogenesis, there are no specific interventions for SIMI.

At the cellular level, cardiomyocyte death is a major manifestation of SIMI [6]. As a kind of iron-dependent-programmed cell death distinguished from apoptosis, pyroptosis, necroptosis, or autophagy-dependent cell death [7], ferroptosis is featured with strong oxidative stress as well as accumulation of lipid peroxides and iron and possesses no typical morphological features [8]. Studies have shown that ferroptosis is engaged in sepsis occurrence and development [9]. Recent research has suggested that ferroptosis is crucial for sepsis-induced organ injury, such as SIMI. [10]. In addition, ferroptosis inhibitor can also improve SIMI and reduces cardiomyocyte death [11]. Consequently, inhibition of cardiomyocyte ferroptosis is an effective approach to protect against SIMI.

Annexin A1 (ANXA1), a glucocorticoid-regulated protein widely expressed in human tissues and cells [12], has been demonstrated to participate in a number of cellular processes, including cell proliferation, differentiation, and apoptosis [13]. As evidenced by prior studies, ANXA1 exerts potent antioxidative and anti-inflammatory effects [14]. Moreover, ANXA1 also plays an advantageous role in cardiovascular diseases [15]. ANXA1 small peptide (ANXA1sp), a bioactive peptide derived from ANXA1, exhibits biological effects similar to those of ANXA1 [16], hinting that ANXA1sp also plays a cardioprotective role in cardiovascular diseases. However, whether ANXA1sp can alleviate SIMI through inhibiting ferroptosis has not been identified yet.

Herein, we aimed to explore whether ANXA1sp alleviates LPS-induced cardiomyocyte damage and elucidates the underlying mechanism, hoping to find a novel therapeutic agent for SIMI treatment.

## 2. Materials and Methods

### 2.1. Cell Culture and Transfection

Murine cardiomyocytes (H9C2 cells) were obtained from Bena Culture Collection (Beijing, China) and cultured in DMEM (10% FBS) in a humid atmosphere (37°C; 5% CO_2_).

Small interfering RNA (siRNA) against SIRT3 (si-SIRT3) and negative control (si-NC) was synthesized by RiboBio (Guangzhou, China). Then, si-NC or si-SIRT3 was transfected into H9C2 cells via Lipofectamine 3000 (Invitrogen). According to a previous study [17], the interference sequences for si-SIRT3 and si-NC are 5′-CAGCAAGGTTCTTACTACA-3′ and 5′-GGCTCTAGAAAAGCCTATGC-3′, respectively.

### 2.2. Cell Treatment

To establish a SIMI model *in vitro*, H9C2 cells were exposed to LPS (1 *μ*g/mL). ANXA1sp applied in this study was synthesized by GenScript Biotech (Piscataway, USA) [18]. For ANXA1sp pretreatment, H9C2 cells were subject to at 0, 10, or 20 *μ*M for 1 h before LPS treatment. Erastin (10 *μ*M) was applied to induce ferroptosis 8 h before LPS treatment [19].

### 2.3. Cell Survival

Cell survival was evaluated via CCK-8 and LDH assays. In brief, H9C2 cells were seeded into a 96-well plate (2 × 10^4^ cells/well) and exposed to LPS for 24 h. Then, the CCK-8 reagent was added (10 *μ*L/well). H9C2 cells were cultured with CCK-8 reagent at 37°C for another 2 h. The absorbance was measured with a microplate reader at 450 nm. LDH release was evaluated via an LDH assay kit (Beyotime, China) as per the standard instruction.

### 2.4. ELISA

ELISA was used to determine tumor necrosis factor- (TNF-) *α*, interleukin- (IL-) 1*β*, and interleukin- (IL-) 6 levels in H9C2 cell supernatants as per the manufacturer's instructions.

### 2.5. Intracellular ROS Assay

To detect ROS content in H9C2 cells, a Reactive Oxygen Species Assay Kit (Beyotime, China) was applied. In brief, H9C2 cells were plated into 6-well plates (1 × 10^5^ cells/well) for 24 h. Then, H9C2 cells were incubated with a DCFH-DA probe, washed, harvested, and resuspended in ice-cold PBS. Finally, the ROS fluorescence was measured via flow cytometry (FACSCalibur, USA).

### 2.6. Measurement of Oxidative Stress-Related Indicators

Briefly, H9C2 cells were lysed in PBS via sonication and centrifuged at 15000× g for 5 min at 4°C to acquire cell supernatants for further measurement. Malondialdehyde (MDA) level, superoxide dismutase (SOD) activity, and glutathione (GSH) level were measured via commercial kits (Nanjing Jiancheng Bioengineering Institute, Nanjing City, China) as per standard instructions.

### 2.7. Iron Assay

Total iron content was measured with an iron assay kit (Sigma-Aldrich). Briefly, H9C2 cells were lysed in the iron assay buffer and centrifuged at 16,000 g for 10 min. Then, 5 *μ*L iron reducer was added to 100 *μ*L cell lysate for total iron (Fe^2+^ and Fe^3+^) assessment. Next, 100 *μ*L iron probe was added. 1 h later, the absorbance was detected at 593 nm via spectrophotometry.

### 2.8. Western Blotting

Proteins were extracted from H9C2 cells, separated by SDS-PAGE, and transferred onto PVDF membranes. Then, the membranes were blocked with 5% skimmed milk at room temperature. Next, the membranes were incubated with primary antibodies (anti-GPX4 antibody (1 : 2000 dilution; ab125066; Abcam, Cambridge, UK), anti-FTH1 antibody (1 : 2000 dilution; ab75972; Abcam), anti-SIRT3 antibody (1 : 2000 dilution; ab246522; Abcam), anti-p53 antibody (1 : 2000 dilution; ab26; Abcam), anti-ac-p53 antibody (1 : 2000 dilution; 2570S; Cell Signaling Technology, Danvers, USA), and anti-GAPDH antibody (1 : 10000 dilution; ab9485; Abcam)) at 4°C overnight and HRP-conjugated secondary antibodies (1 : 5000 dilution; ab6721 and ab6728; Abcam) at room temperature for 1 h. An ECL chemiluminescence detection system was adopted to visualize the target bands.

### 2.9. Coimmunoprecipitation (Co-IP)

Co-IP was performed using indicated antibodies and IgG (Invitrogen) as per standard instructions. Briefly, H9C2 cell lysates were incubated with antibody-conjugated beads at 4°C for 2 h. Then, the beads were collected, washed extensively, and boiled in an SDS loading buffer. Western blot was applied to study the immunoprecipitated proteins.

### 2.10. Statistical Analysis

GraphPad 6.0 software was utilized for statistical analysis. All data were displayed as mean ± SD. Comparisons were performed with Student's *t*-test (2 groups) and one-way analysis of variance (ANOVA) (>2 groups). Differences with *p* < 0.05 were deemed statistically significant.

## 3. Results

### 3.1. ANXA1sp Inhibits LPS-Induced H9C2 Cell Damage

**Figure 1:**
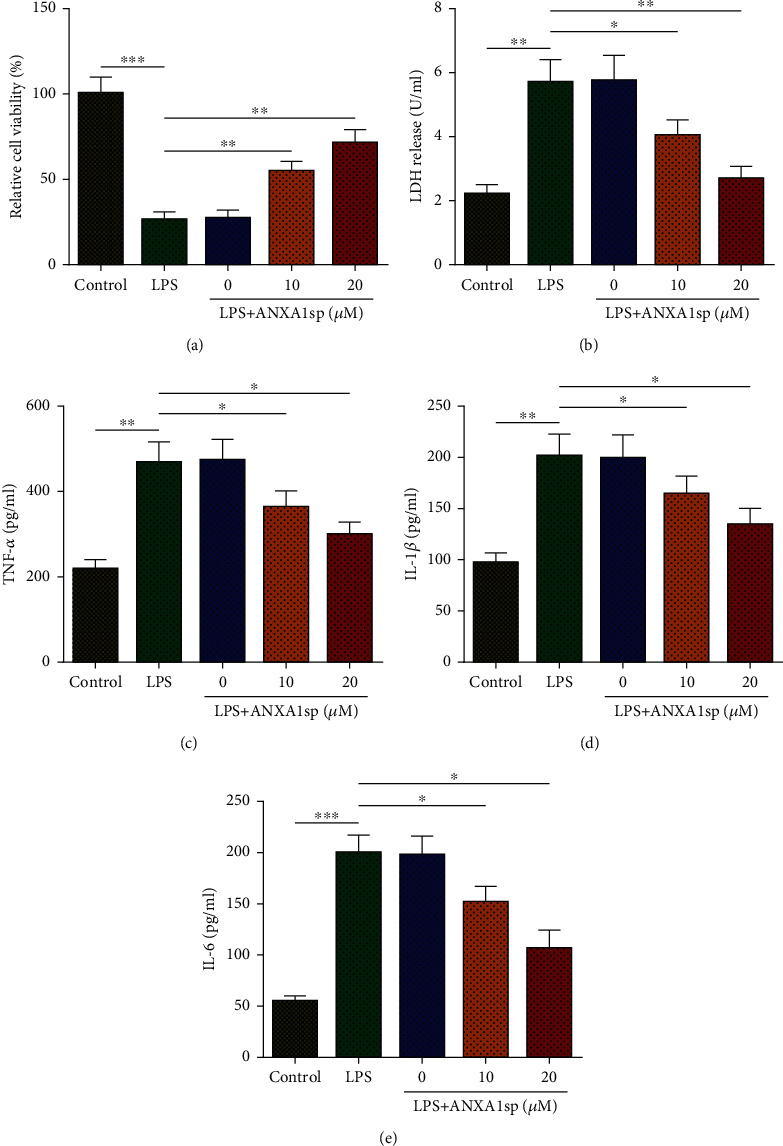
ANXA1sp inhibits LPS-induced H9C2 cell damage. H9C2 cells were assigned to control, LPS, and LPS+ANXA1sp groups. (a) CCK-8 for H9C2 cell viability. (b) LDH level in H9C2 cell supernatant. (c–e) ELISA for TNF-*α*, IL-1*β*, and IL-6 levels in H9C2 cell supernatant. The experiments were repeated three times (*n* = 3). Data were presented as mean ± SD. All statistical analyses were performed using Student's *t*-test or one-way ANOVA. ^∗^*p* < 0.05; ^∗∗^*p* < 0.01; ^∗∗∗^*p* < 0.001.

To simulate SIMI *in vitro*, H9C2 cells were subject to LPS (1 *μ*g/mL). To explore the effects of ANXA1sp on LPS-induced cell damage, H9C2 cells were exposed to ANXA1sp (0, 10, or 20 *μ*M) for 1 h before LPS treatment. CCK-8 assay showed that LPS significantly reduced H9C2 cell vitality, while ANXA1sp remarkably eliminated this effect (Figure 1(a)). Similarly, LDH assay exhibited that LPS-induced LDH release in H9C2 cells could be abated by ANXA1sp pretreatment (Figure 1(b)). Furthermore, ELISA assay showed that LPS caused a remarkable increase in TNF-*α*, IL-1*β*, and IL-6 levels in H9C2 cell supernatant, while ANXA1sp addition abated such an effect (Figures 1(c)–1(e)). Therefore, ANXA1sp could improve LPS-induced cell death and inflammation in H9C2 cardiomyocytes.

### 3.2. ANXA1sp Inhibits LPS-Caused Oxidative Stress and Ferroptosis in H9C2 Cardiomyocytes

**Figure 2:**
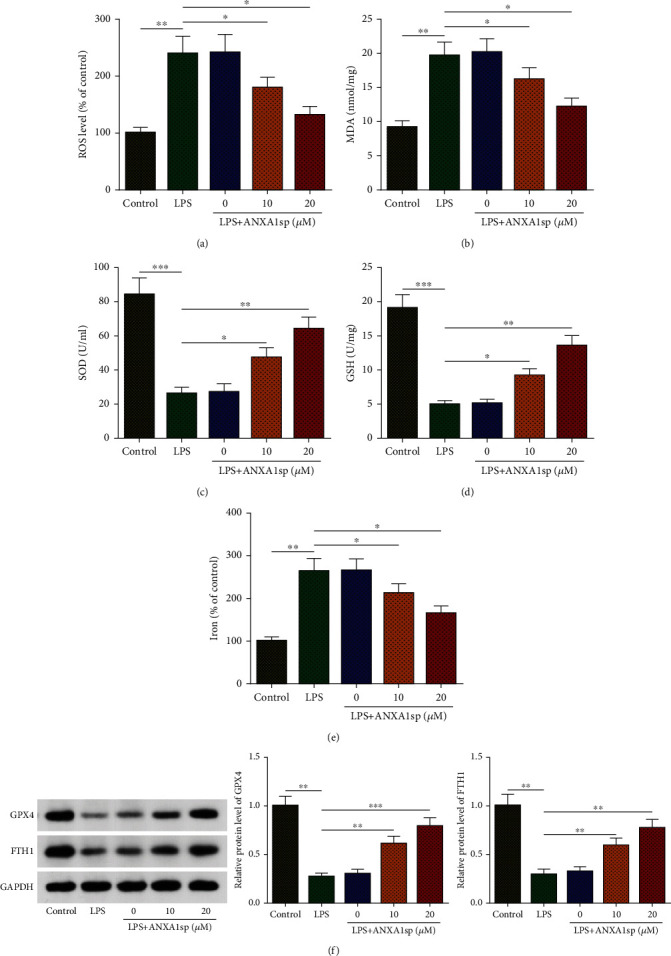
ANXA1sp inhibits LPS-caused oxidative stress and ferroptosis in H9C2 cardiomyocytes. H9C2 cells were assigned to control, LPS, and LPS+ANXA1sp groups. (a–d) ROS, MDA, SOD, and GSH levels in H9C2 cell supernatant. (e) Iron accumulation in H9C2 cells. (f) WB for levels of ferroptosis-related proteins (GPX4 and FTH1) in H9C2 cells. The experiments were repeated three times (*n* = 3). Data were presented as mean ± SD. All statistical analyses were performed using Student's *t*-test or one-way ANOVA. ^∗^*p* < 0.05; ^∗∗^*p* < 0.01; ^∗∗∗^*p* < 0.001.

ANXA1sp has been proven to protect against oxidative injury [18]. As illustrated in Figures 2(a)–2(d), LPS caused a substantial increase in ROS generation and MDA level but a significant decrease in SOD activity and GSH (Figure 2(d)) level, which was substantially reversed by ANXA1sp pretreatment, suggesting that ANXA1sp relieved LPS-induced oxidative stress damage in H9C2 cells.

In order to evaluate whether ANXA1sp affects LPS-induced ferroptosis of cardiomyocytes, iron accumulation and ferroptosis-related factors (GPX4 and FTH1) were assessed. It was shown that LPS increased iron content and decreased GPX4 and FTH1 protein levels in H9C2 cells; however, ANXA1sp partly eliminated such effects, indicating the inhibitory effect of ANXA1sp on LPS-induced ferroptosis in H9C2 cells (Figures 2(e) and 2(f)). Taken together, our data revealed that ANXA1sp repressed oxidative stress as well as ferroptosis in LPS-challenged H9C2 cardiomyocytes.

### 3.3. Erastin Eliminates the Protective Effect of ANXA1sp against LPS-Induced H9C2 Cell Death, Inflammation, and Oxidative Stress

**Figure 3:**
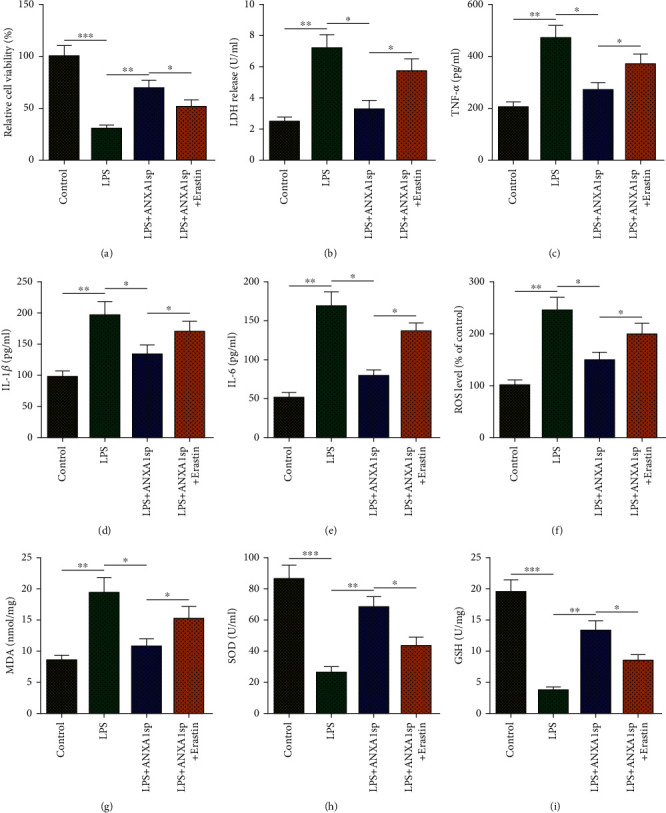
Erastin eliminates the protective effect of ANXA1sp against LPS-induced H9C2 cell death, inflammation, and oxidative stress. H9C2 cells were assigned to control, LPS, LPS+ANXA1sp, and LPS+ANXA1sp+erastin groups. (a) CCK-8 for H9C2 cell viability in each group. (b) LDH level in H9C2 cell supernatant from each group. (c–e) ELISA for TNF-*α*, IL-1*β*, and IL-6 levels in H9C2 cell supernatant. (f–i) ROS, MDA, SOD, and GSH levels in H9C2 cell supernatant. The experiments were repeated three times (*n* = 3). Data were presented as mean ± SD. All statistical analyses were performed using Student's *t*-test or one-way ANOVA. ^∗^*p* < 0.05; ^∗∗^*p* < 0.01; ^∗∗∗^*p* < 0.001.

In order to confirm whether ANXA1sp relieves LPS-induced H9C2 cell damage by inhibiting ferroptosis, erastin (10 *μ*M), a ferroptosis agonist, was applied to induce H9C2 cell ferroptosis 8 h before LPS treatment. Results of the CCK-8 assay exhibited that erastin eliminated ANXA1sp-mediated improvement of the cell viability of LPS-triggered H9C2 cells (Figure 3(a)). LDH assay further affirmed that the suppressive effect of ANXA1sp on LPS-induced H9C2 death was abated by erastin (Figure 3(b)). In addition, erastin treatment reversed ANXA1sp-induced repressive effects on the inflammatory response of LPS-challenged H9C2 cells, which was manifested as the increase in TNF-*α*, IL-1*β*, and IL-6 levels (Figures 3(c)–3(e)). Moreover, erastin also abated ANXA1sp-mediated alleviation of LPS-induced oxidative stress in H9C2 cells (Figures 3(f)–3(i)). The above results indicated that ANXA1sp inhibited LPS-induced H9C2 cardiomyocyte damage by inhibiting ferroptosis.

### 3.4. ANXA1sp Promotes p53 Deacetylation by Enhancing SIRT3 Expression

**Figure 4:**
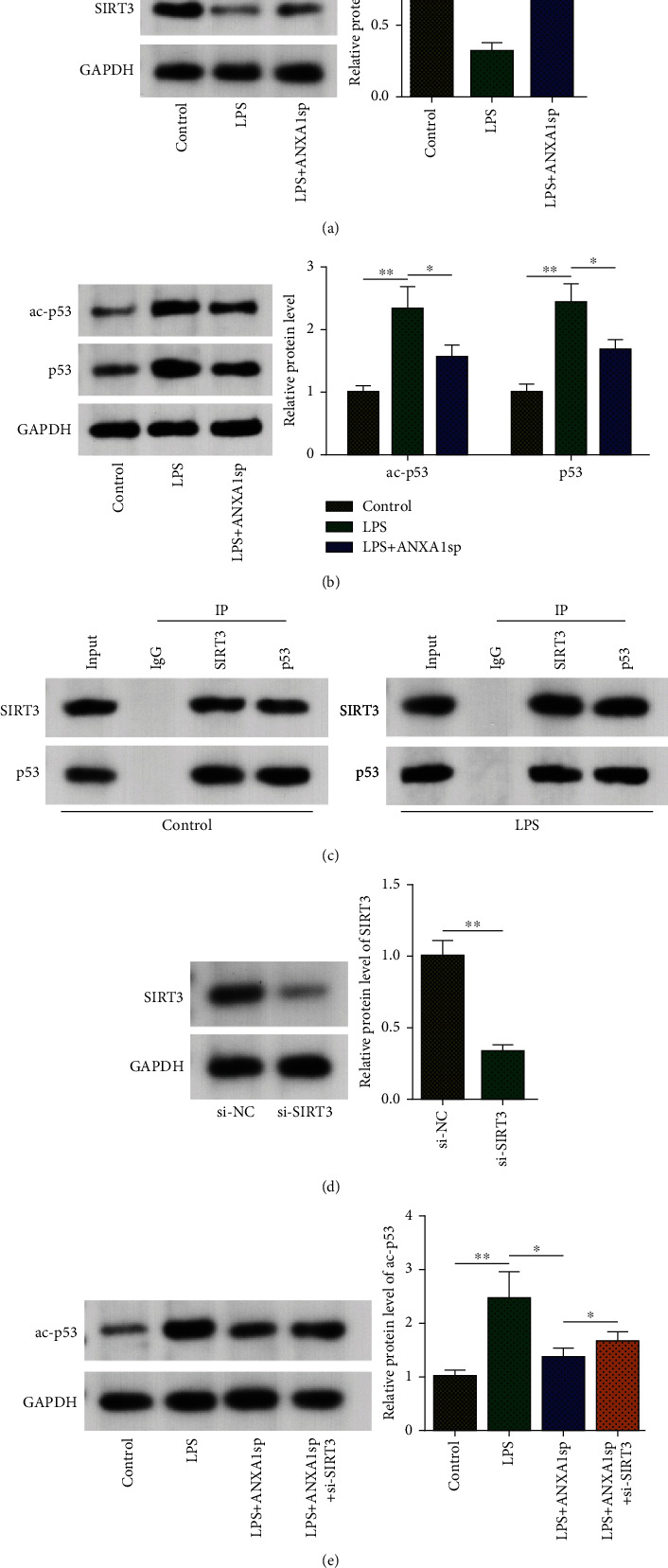
ANXA1sp promotes p53 deacetylation by enhancing SIRT3 expression. (a, b) WB for SIRT3, ac-p53, and p53 protein levels in H9C2 cells from control, LPS, or LPS+ANXA1sp groups. (c) Co-IP for interaction between SIRT3 and p53 in H9C2 cells from control or LPS groups. (d) Western blotting for H9C2 cells transfected with si-NC or si-SIRT3. (e) Western blotting for ac-p53 protein level in H9C2 cells from control, LPS, LPS+ANXA1sp, or LPS+ANXA1sp+si-SIRT3 groups. The experiments were repeated three times (*n* = 3). Data were presented as mean ± SD. All statistical analyses were performed using Student's t-test or one-way ANOVA. ^∗^*p* < 0.05; ^∗∗^*p* < 0.01; ^∗∗∗^*p* < 0.001.

Since sirtuin 3 (SIRT3) exerts cardioprotective effects in cardiovascular diseases, including SIMI [20–22], SIRT3 expression in H9C2 cells was detected. It was revealed that the SIRT3 protein level decreased under LPS stimulation but increased after ANXA1sp treatment (Figure 4(a)), suggesting ANXA1sp increased SIRT3 expression in LPS-induced H9C2 cells. Studies have indicated that p53 acetylation is deeply involved in SIMI [23]. In addition, p53 acetylation also inhibits ferroptosis [24]. Interestingly, LPS significantly upregulated the protein expression level and acetylation level of p53 in H9C2 cells, which was reversed by ANXA1sp (Figure 4(b)), indicating ANXA1sp inhibited p53 expression and acetylation in LPS-triggered H9C2 cells.

As a deacetylase, SIRT3 has been identified as a regulator of p53 acetylation [25]. Therefore, we hypothesized that a similar mechanism might be involved in SIMI. To investigate whether ANXA1sp could affect p53 deacetylation via SIRT3, the binding of SIRT3 with p53 was first assessed by coimmunoprecipitation. As shown in Figure 4(c), SIRT3 could coimmunoprecipitate with p53 in H9C2 cells. Then, SIRT3 was knocked down in H9C2 cells with transfection efficiency assessed by western blotting (Figure 4(d)). Moreover, ANXA1sp pretreatment significantly reduced the acetylated p53 level in H9C2 cells, while SIRT3 knockdown substantially reversed such an effect (Figure 4(e)). Thus, it could be concluded that ANXA1sp coffered p53 deacetylation by upregulating SIRT3 expression in LPS-treated H9C2 cardiomyocytes.

### 3.5. SIRT3 Inhibition Attenuates the Protective Effects of ANXA1sp on LPS-Challenged H9C2 Cells

**Figure 5:**
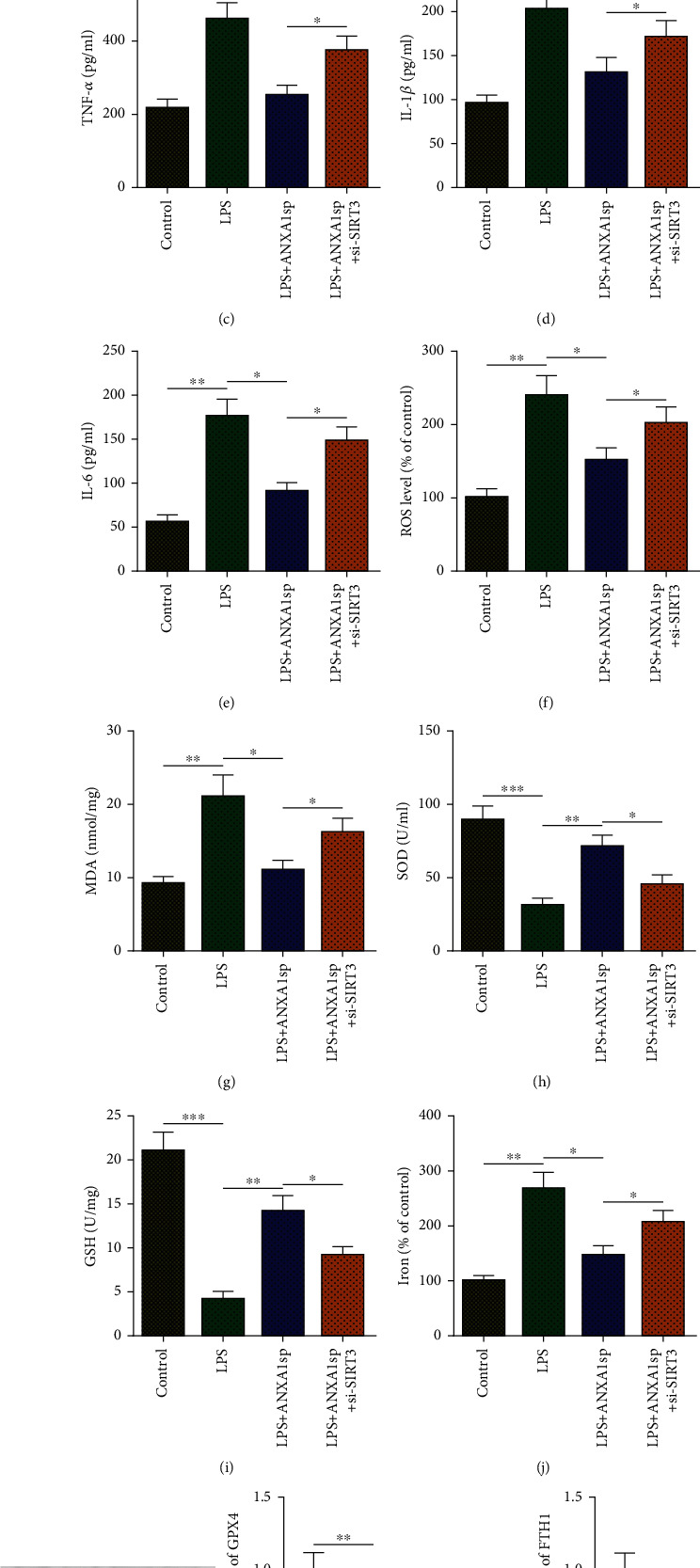
SIRT3 inhibition attenuates the protective effects of ANXA1sp on LPS-challenged H9C2 cells. H9C2 cells were assigned to control, LPS, LPS+ANXA1sp, and LPS+ANXA1sp+si-SIRT3 groups. (a) CCK-8 for H9C2 cell viability. (b) LDH level in H9C2 cell supernatant. (c–e) ELISA for TNF-*α*, IL-1*β*, and IL-6 levels. (f–i) ROS, MDA, SOD, and GSH levels in H9C2 cell supernatant. (j) Iron accumulation in H9C2 cells. (k) WB for levels of ferroptosis-related proteins (GPX4 and FTH1). The experiments were repeated three times (*n* = 3). Data were presented as mean ± SD. All statistical analyses were performed using Student's *t*-test or one-way ANOVA. ^∗^*p* < 0.05; ^∗∗^*p* < 0.01; ^∗∗∗^*p* < 0.001.

To further verify whether the alleviative effects of ANXA1sp depend on SIRT3, H9C2 cells were assigned to control, LPS, LPS+ANXA1sp, and LPS+ANXA1sp+si-SIRT3 groups. As illustrated by CCK-8 and LDH assays, ANXA1sp-mediated inhibition of cell death of LPS-treated H9C2 cells was significantly blocked when SIRT3 was knockdown (Figures 5(a) and 5(b)). Consequently, SIRT3 deletion reversed the inhibitory effects of ANXA1sp on TNF-*α*, IL-1*β*, and IL-6 levels in LPS-challenged H9C2 cells (Figures 5(c)–5(e)). Additionally, ANXA1sp failed to rescue LPS-triggered oxidative stress in H9C2 cells in the presence of SIRT3 silencing (Figures 5(f)–5(i)). Furthermore, SIRT3 inhibition significantly abated ANXA1sp-mediated increase in total iron content as well as GPX4 and FTH1 protein levels in LPS-triggered H9C2 cells (Figures 5(j) and 5(k)). Therefore, the alleviative effects of ANXA1sp on LPS-induced H9C2 cells relied on SIRT3 upregulation.

## 4. Discussion

The effects of ANXA1sp on the suppression of inflammatory response [26] and oxidative injury [18] have been substantiated in previous research. Moreover, one study has demonstrated that ANXA1sp could also alleviate myocardial inflammation [27], implying that ANXA1sp plays a protective role in cardiac disorders. However, whether ANXA1sp could improve SIMI and the underlying mechanisms involved in LPS-caused H9C2 cardiomyocyte damage was still unclear. Our findings demonstrated that ANXA1sp inhibited ferroptosis-induced cell damage in LPS-challenged H9C2 cardiomyocytes via SIRT3-dependent p53 deacetylation, indicating that ANXA1sp could improve SIMI *in vitro*.

As a reversible form of cardiac depression, SIMI is featured with extravagant inflammatory response, oxidative stress, and cardiomyocyte death [28, 29]. In this study, H9C2 cardiomyocytes were subject to LPS treatment to simulate SIMI *in vitro*. To explore the effects of ANXA1sp on H9C2 cells, H9C2 cells were pretreated with ANXA1sp before LPS treatment. It was shown that LPS exposure led to increased H9C2 cell death and LDH release, exorbitant secretion of proinflammatory factors, and enhanced oxidative stress response; however, ANXA1sp administration conspicuously inhibited LPS-induced H9C2 cell damage in a dose-dependent manner.

There is a growing body of evidence that ferroptosis has a role in a number of cardiovascular conditions, including heart failure, myocardial infarction, stroke, and arrhythmia [30], suggesting ferroptosis inhibition could be a potential therapeutic approach for cardiovascular diseases. Additionally, ferroptosis plays a significant role in controlling oxidative stress and inflammatory responses in cardiovascular diseases [31]. Also, ferroptosis plays a critical role in sepsis-associated organ injury, including SIMI [10]. Taking into account the anti-inflammatory and antioxidant effect of ANXA1sp, it was conjectured that the effects of ANXA1sp on LPS-caused H9C2 cardiomyocyte damage may involve ferroptosis. As expected, ANXA1sp partly relieved LPS-triggered H9C2 cell ferroptosis. Additionally, erastin, a ferroptosis agonist, could eliminate the protective effects of ANXA1sp on LPS-induced H9C2 cardiomyocyte death, inflammation, and oxidative stress, indicating that ANXA1sp-mediated protection against LPS-challenged H9C2 cells was achieved by inhibiting H9C2 cell ferroptosis.

SIRT3, a soluble protein located in the mitochondria, is a histone deacetylase that regulates cell metabolism through the deacetylation of a variety of protein substrates [25]. Previous research has demonstrated that SIRT3 expression considerably decreased in myocardial tissues of mice with LPS-induced myocardial injury, while restoration of SIRT3 expression could prevent LPS-induced myocardial injury [32]. The antioxidative and anti-inflammation effects on SIRT3 in LPS-challenged cardiomyocytes have also been demonstrated [33]. Intriguingly, Suliman et al. found that ANXA1sp could upregulate SIRT3 expression in the mitochondria of kidney tubular cells [18]. In line with the above studies, SIRT3 expression markedly declined in LPS-triggered H9C2 cells; however, ANXA1sp treatment distinctly restored SIRT3 expression. Furthermore, SIRT3 silence could eliminate the impact of ANXA1sp pretreatment on LPS-triggered H9C2 cardiomyocyte death, inflammation, oxidative stress, and ferroptosis.

The p53 protein is widely recognized as a “guardian of the genome” for its essential role in maintaining genomic stability [34]. As a critical regulator of cellular processes, p53 is engaged in a variety of cellular processes, such as cell differentiation, senescence, proliferation, apoptosis, ferroptosis, and autophagy [35]. Former studies have proven that p53 deacetylation alleviates sepsis-induced organ damage [36, 37]. Also, p53 deacetylation could inhibit ferroptosis [24]. Additionally, there is evidence that SIRT3 activates p53 deacetylation in H9C2 cells [38]. Consistently, it was found that SIRT3 could coimmunoprecipitate with p53 in H9C2 cells. Moreover, ANXA1sp pretreatment significantly attenuated the LPS-induced increase of p53 expression and acetylation in H9C2 cells, which was substantially reversed by SIRT3 silencing. Taken together, ANXA1sp contributed to p53 deacetylation in LPS-treated H9C2 cells by enhancing SIRT3 expression.

## 5. Conclusion

In summary, this study uncovered that ANXA1sp could inhibit LPS-induced cytotoxicity in H9C2 cardiomyocytes. Moreover, it was also demonstrated that ANXA1sp-mediated alleviation of LPS-triggered H9C2 cardiomyocyte death, inflammation, oxidative stress, and ferroptosis was closely related to SIRT3-dependent p53 deacetylation. These findings indicated that ANXA1sp might be a novel therapeutic agent for the treatment of SIMI. However, the protective effects of ANXA1sp against SIMI need further validation *in vivo*.

## Data Availability

The data used to support the findings of this study are available from the corresponding author upon request.

## References

[1] Angeletti S., Dicuonzo G., Fioravanti M. (2015). Procalcitonin, MR-proadrenomedullin, and cytokines measurement in sepsis diagnosis: advantages from test combination. *Disease Markers*.

[2] van der Poll T. (2016). Future of sepsis therapies. *Critical Care*.

[3] Meng Z. J., Wang C., Meng L. T., Bao B. H., Wu J. H., Hu Y. Q. (2018). Sodium tanshinone IIA sulfonate attenuates cardiac dysfunction and improves survival of rats with cecal ligation and puncture-induced sepsis. *Chinese Journal of Natural Medicines*.

[4] Wilhelm J., Hettwer S., Schuermann M. (2013). Severity of cardiac impairment in the early stage of community-acquired sepsis determines worse prognosis. *Clinical Research in Cardiology*.

[5] Li Y., Zhao Y., Qiu C. (2020). Role of eotaxin-1/CCL11 in sepsis-induced myocardial injury in elderly patients. *Aging*.

[6] Durand A., Duburcq T., Dekeyser T. (2017). Involvement of mitochondrial disorders in septic cardiomyopathy. *Oxidative Medicine and Cellular Longevity*.

[7] Dixon S. J., Lemberg K. M., Lamprecht M. R. (2012). Ferroptosis: an iron-dependent form of nonapoptotic cell death. *Cell*.

[8] Yang Y. C., Zhang M. Y., Liu J. Y., Jiang Y. Y., Ji X. L., Qu Y. Q. (2022). Identification of ferroptosis-related hub genes and their association with immune infiltration in chronic obstructive pulmonary disease by bioinformatics analysis. *International Journal of Chronic Obstructive Pulmonary Disease*.

[9] Liu Y., Tan S., Wu Y., Tan S. (2022). The emerging role of ferroptosis in sepsis. *DNA and Cell Biology*.

[10] Li N., Wang W., Zhou H. (2020). Ferritinophagy-mediated ferroptosis is involved in sepsis-induced cardiac injury. *Free Radical Biology & Medicine*.

[11] Xiao Z., Kong B., Fang J. (2021). Ferrostatin-1 alleviates lipopolysaccharide-induced cardiac dysfunction. *Bioengineered*.

[12] Purvis G. S. D., Solito E., Thiemermann C. (2019). Annexin-A1: therapeutic potential in microvascular disease. *Frontiers in Immunology*.

[13] Yap G. L. R., Sachaphibulkij K., Foo S. L., Cui J., Fairhurst A. M., Lim L. H. K. (2020). Annexin-A1 promotes RIG-I-dependent signaling and apoptosis via regulation of the IRF3-IFNAR-STAT1-IFIT1 pathway in A549 lung epithelial cells. *Cell Death & Disease*.

[14] Yu C., Zhang L. (2022). Methylprednisolone up-regulates annexin A1 (ANXA1) to inhibit the inflammation, apoptosis and oxidative stress of cigarette smoke extract (CSE)-induced bronchial epithelial cells, a chronic obstructive pulmonary disease in vitro model, through the formyl peptide receptor 2 (FPR2) receptors and the adenosine 5'-monophosphate (AMP)-activated protein kinase (AMPK) pathway. *Bioengineered*.

[15] de Jong R., Leoni G., Drechsler M., Soehnlein O. (2017). The advantageous role of annexin A1 in cardiovascular disease. *Cell Adhesion & Migration*.

[16] Sheikh M. H., Solito E. (2018). Annexin A1: uncovering the many talents of an old protein. *International Journal of Molecular Sciences*.

[17] Peng S., Lu X. F., Qi Y. D. (2020). LCZ696 ameliorates oxidative stress and pressure overload-induced pathological cardiac remodeling by regulating the Sirt3/MnSOD pathway. *Oxidative Medicine and Cellular Longevity*.

[18] Suliman H., Ma Q., Zhang Z. (2021). Annexin A1 tripeptide mimetic increases sirtuin-3 and augments mitochondrial function to limit ischemic kidney injury. *Frontiers in Physiology*.

[19] Ma S., Sun L., Wu W., Wu J., Sun Z., Ren J. (2020). USP22 protects against myocardial ischemia-reperfusion injury via the SIRT1-p53/SLC7A11-dependent inhibition of ferroptosis-induced cardiomyocyte death. *Frontiers in Physiology*.

[20] Xin T., Lu C. (2020). SirT3 activates AMPK-related mitochondrial biogenesis and ameliorates sepsis-induced myocardial injury. *Aging*.

[21] Palomer X., Román-Azcona M. S., Pizarro-Delgado J. (2020). SIRT3-mediated inhibition of FOS through histone H3 deacetylation prevents cardiac fibrosis and inflammation. *Signal Transduction and Targeted Therapy*.

[22] Yu W., Gao B., Li N. (2017). Sirt3 deficiency exacerbates diabetic cardiac dysfunction: role of Foxo3A-Parkin-mediated mitophagy. *Biochimica et Biophysica Acta - Molecular Basis of Disease*.

[23] Han D., Li X., Li S. (2017). Reduced silent information regulator 1 signaling exacerbates sepsis-induced myocardial injury and mitigates the protective effect of a liver X receptor agonist. *Free Radical Biology & Medicine*.

[24] Chen H., Lin X., Yi X. (2022). SIRT1-mediated p53 deacetylation inhibits ferroptosis and alleviates heat stress-induced lung epithelial cells injury. *International Journal of Hyperthermia*.

[25] Chen J., Wang A., Chen Q. (2017). SirT3 and p53 deacetylation in aging and cancer. *Journal of Cellular Physiology*.

[26] Zhang Z., Ma Q., Shah B. (2017). Neuroprotective effects of annexin A1 tripeptide after deep hypothermic circulatory arrest in rats. *Frontiers in Immunology*.

[27] Ma Q., Zhang Z., Shim J. K. (2019). Annexin A1 bioactive peptide promotes resolution of neuroinflammation in a rat model of exsanguinating cardiac arrest treated by emergency preservation and resuscitation. *Frontiers in Neuroscience*.

[28] Zhong J., Tan Y., Lu J. (2019). Therapeutic contribution of melatonin to the treatment of septic cardiomyopathy: a novel mechanism linking Ripk3-modified mitochondrial performance and endoplasmic reticulum function. *Redox Biology*.

[29] Mao Y., Ren J., Yang L. (2022). FUN14 domain containing 1 (FUNDC1): a promising mitophagy receptor regulating mitochondrial homeostasis in cardiovascular diseases. *Frontiers in Pharmacology*.

[30] Huang F., Yang R., Xiao Z. (2021). Targeting ferroptosis to treat cardiovascular diseases: a new continent to be explored. *Frontiers in Cell and Development Biology*.

[31] Yu Y., Yan Y., Niu F. (2021). Ferroptosis: a cell death connecting oxidative stress, inflammation and cardiovascular diseases. *Cell Death Discovery*.

[32] Xu Y., Zhang S., Rong J. (2020). Sirt3 is a novel target to treat sepsis induced myocardial dysfunction by acetylated modulation of critical enzymes within cardiac tricarboxylic acid cycle. *Pharmacological Research*.

[33] Cheng Z., Lv D., Luo M. (2021). Tubeimoside I protects against sepsis-induced cardiac dysfunction via SIRT3. *European Journal of Pharmacology*.

[34] Rodrigues M., Antonucci I., Elabd S. (2018). p53 is active in human amniotic fluid stem cells. *Stem Cells and Development*.

[35] Olivos D. J., Mayo L. D. (2016). Emerging non-canonical functions and regulation by p53: p53 and stemness. *International Journal of Molecular Sciences*.

[36] Sun M., Li J., Mao L. (2021). p53 deacetylation alleviates sepsis-induced acute kidney injury by promoting autophagy. *Frontiers in Immunology*.

[37] Zhang W., Zhang Y., Guo X. (2017). Sirt1 protects endothelial cells against LPS-induced barrier dysfunction. *Oxidative Medicine and Cellular Longevity*.

[38] Li L., Zeng H., He X., Chen J. X. (2021). Sirtuin 3 alleviates diabetic cardiomyopathy by regulating TIGAR and cardiomyocyte metabolism. *Journal of the American Heart Association*.

